# ‘Explanting’ the well-fixed, cemented acetabular implant

**DOI:** 10.1308/003588413X13511609958055f

**Published:** 2013-03

**Authors:** H Arshad, N Chirodian

**Affiliations:** Norfolk and Norwich University Hospitals NHS Foundation Trust, UK

## Background

Aseptic loosening is the most common indication for revision of a cemented acetabular implant. Removal of loose components is straightforward. Revision surgery for malposition, infection or polyethylene wear may require the removal of a well-fixed acetabular component. This can be achieved with the use of osteotomes, reamers and drills.^1,2^ We describe a rapid and straightforward technique that may be used as an alternative, as well as discussing its limitations.

## Technique

**Figure 1:**
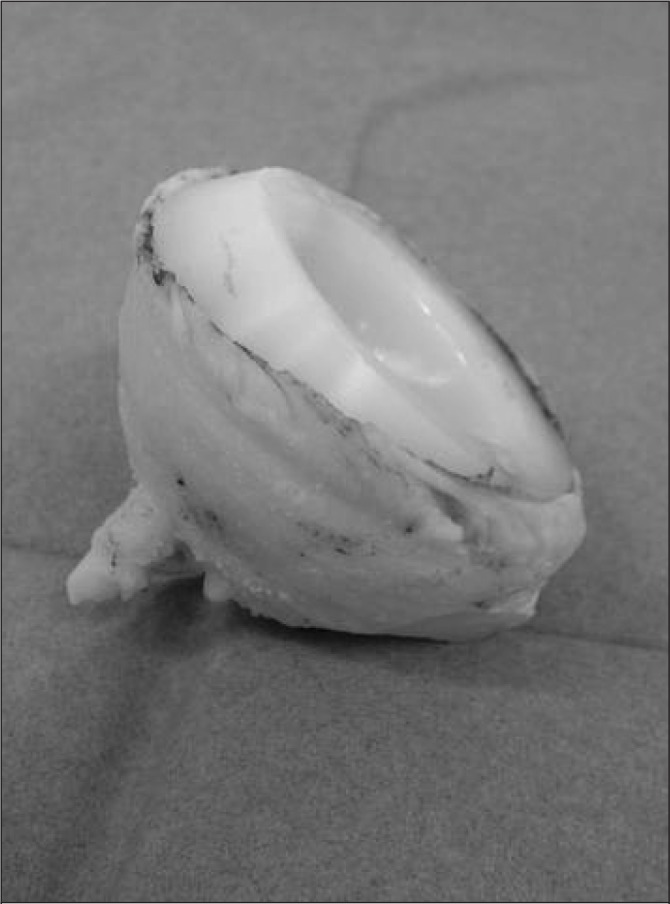
Removed acetabular component

The acetabular rim is exposed as usual. The Explant^®^ Acetabular Cup Removal System (Zimmer, Swindon, UK) is selected, sized 2mm larger than the acetabular component. The blade of the device is pushed into the bone–cement interface and is used to circumscribe first the rim and then the full radius of the cement mantle surrounding the implant. Typically, the process takes less than five minutes and the whole cement mantle emerges, complete with its key holes (Fig 1).

## Discussion

We would caution against use of this technique in certain circumstances. It is difficult to maintain the Explant^®^ seated in a good position if the implant is not well fixed or if there is gross polyethylene wear. We use different techniques where the acetabular wall is thin or the cement mantle is eccentric.
